# Mature B, T and NK-cell, plasma cell and histiocytic/dendritic cell neoplasms: classification according to the World Health Organization and International Consensus Classification

**DOI:** 10.1186/s13045-024-01570-5

**Published:** 2024-07-08

**Authors:** Judith A. Ferry, Brian Hill, Eric D. Hsi

**Affiliations:** 1 Department of Pathology, Massachusetts General Hospital and Harvard Medical School, 55 Fruit Street, Boston, MA 02114 USA; 2 Department of Hematology and Medical Oncology, Cleveland Clinic, Cleveland, OH USA; 3 Department of Laboratory Medicine and Pathology, Mayo Clinic, Rochester, MN USA

## Abstract

In 2022, two updated classification systems for lymphoid neoplasms were published by the World Health Organization (WHO Classification of Haematolymphoid Tumours, 5th edition, referred to hereafter as WHO-HAEM5) and the International Consensus Conference (ICC) (Alaggio et al. in Leukemia 36(7):1720–1748, 2022; Campo et al. in Blood 140(11):1229–1253, 2022). Both classifications were conceived by both pathologists and clinicians with expertise in the field. The reasons for this have been reviewed previously (Arber et al. in Virchows Arch 482(1):1–9, 2023; Cree in Leukemia 36(7):1701–1702, 2022, Leukemia 36(11):2750, 2022). Given that both groups were using data-driven processes and consensus and used the revised 4th edition of the WHO Classification of Haematolymphoid Tumours (WHO-HAEM4R) as a starting point, it is not entirely surprising that the resulting classifications are quite similar. However, they are not identical and reflect preferences or approaches for certain unsettled areas as well as preferred terminology. In this review, we will compare nomenclature of the WHO-HAEM5 and ICC classifications, focusing on lymphoid neoplasms and lymphoproliferative disorders (LPDs).

## Tumor-like lesions with B-cell predominance and tumor-like lesions with T-cell predominance

WHO-HAEM5 has introduced categories of tumor-like lesions with B-cell predominance and T-cell predominance, respectively, not included in WHO-HAEM4R or ICC. The tumor-like lesions with B-cell predominance include reactive B-cell rich lymphoid proliferations that can mimic lymphoma (for example, infectious mononucleosis), IgG4-related disease, and the different types of Castleman disease. The tumor-like lesions with T-cell predominance include Kikuchi-Fujimoto disease, autoimmune lymphoproliferative syndrome and indolent T lymphoblastic proliferation.

## Mature B-cell lymphomas, leukemias and lymphoproliferative disorders

The classification of mature B-cell lymphomas, leukemias and lymphoproliferative disorders in the most recent World Health Organization (WHO-HAEM5) classification of hematolymphoid neoplasms and the International Consensus Classification (ICC) is similar, with many diagnostic categories having identical nomenclature. There is a subset of lymphoid neoplasms in which the two classifications differ, and both show some modifications of the prior WHO classification (WHO-HAEM4R). This review highlights the similarities and the differences among WHO-HAEM4R, WHO-HAEM5 and ICC (Tables 1, 2, 3, 4, 5).

**Table 1:** Comparison of WHO-HAEM4R, ICC and WHO-HAEM5 Classification Mature B-cell lymphomas, leukemias and lymphoproliferative disorders

WHO-HAEM4R	International Consensus Conference (ICC)	WHO-HAEM5
Mature B-cell lymphomas, leukemias and lymphoproliferative disorders		
Chronic lymphocytic leukemia, small lymphocytic lymphoma, B-cell prolymphocytic leukemia and monoclonal B lymphocytosis		
Chronic lymphocytic leukemia/small lymphocytic lymphoma (CLL/SLL)	CLL/SLL	CLL/SLL
Monoclonal B-cell lymphocytosis (MBL) CLL type Non-CLL type	MBL CLL type Non-CLL type	MBL CLL type Non-CLL type
B-Prolymphocytic leukemia (B-PLL)	B-PLL	Category removed
**Splenic B cell lymphomas/leukemias**		
Hairy cell leukemia	Hairy cell leukemia	Hairy cell leukemia
Splenic MZL (SMZL)	SMZL	SMZL
*Splenic B-cell leukemia lymphoma, unclassifiable*	*Splenic B-cell leukemia lymphoma, unclassifiable*	
*Splenic diffuse red pulp small B-cell lymphoma* *Hairy cell leukemia-variant*	*Splenic diffuse red pulp small B-cell lymphoma* *Hairy cell leukemia-variant*	Splenic diffuse red pulp small B-cell lymphoma Splenic B-cell lymphoma/leukemia with prominent nucleoli
**Lymphoplasmacytic lymphoma**		
Lymphoplasmacytic lymphoma (LPL)	LPL	LPL
**Marginal zone lymphomas**		
Extranodal marginal zone lymphoma of mucosa-associated lymphoid tissue (EMZL)	EMZL	EMZL
Primary cutaneous MZL (included under EMZL)	Primary cutaneous marginal zone lymphoproliferative disorder	Primary cutaneous MZL
Nodal MZL *-*Pediatric MZL	Nodal MZL -Pediatric nodal MZL	Nodal MZL
		Pediatric nodal MZL
**Follicular lymphoma**		
Follicular lymphoma (FL) (FL, grades 1-3A, 3B) In situ follicular neoplasia Duodenal-type FL Testicular FL Pediatric type FL Primary cutaneous follicle center lymphoma Also recognized are rare, largely diffuse cases, often inguinal, lacking BCL2 rearrangement and expressing CD23 with loss at 1p36	FL (FL, grade 1-3A, 3B) In situ follicular neoplasia Duodenal-type FL * BCL2-R* negative, CD23 + follicle center lymphoma Primary cutaneous follicle center lymphoma Pediatric type FL Testicular FL	FL Classic FL (cFL; grading [1-3A] is optional) Follicular large B-cell lymphoma FL with unusual cytologic features (blastoid or large centrocyte variant cytologic features) FL with predominantly diffuse growth pattern In situ follicular B-cell neoplasm Duodenal-type FL Pediatric type FL Primary cutaneous follicle center lymphoma
**Mantle cell lymphoma**		
Mantle cell lymphoma (MCL) In situ MCL Non-nodal MCL	MCL In situ MCL Leukemic non-nodal MCL	MCL In situ mantle cell neoplasm Leukemic non-nodal MCL
**Large B-cell lymphomas**		
Diffuse large B-cell lymphoma, not otherwise specified (DLBCL, NOS) *Molecular subtypes* Germinal center B-cell subtype Activated B-cell subtype *Morphological variants* Centroblastic Immunoblastic Anaplastic Other rare variants	DLBCL, NOS Germinal center B-cell type Activated B-cell type	DLBCL, NOS Germinal center B-cell subtype Activated B-cell subtype Centroblastic subtype Immunoblastic subtype Anaplastic subtype DLBCL with *MYC* and *BCL6* rearrangements
T-cell/histocyte rich large B-cell lymphoma (TCHRLBCL)	TCHRLBCL	TCHRLBCL
High grade B-cell lymphoma (HGBCL) with *MYC* and *BCL2* and/or *BCL6* rearrangement	HGBL with *MYC* and *BCL2* rearrangement *HGBL with MYC and BCL6 rearrangement*	DLBCL/HGBL with *MYC* and *BCL2* rearrangement (see DLBCL, NOS)
ALK + LBCL	ALK + LBCL	ALK + LBCL
Large B-cell lymphoma with IRF4 rearrangement	Large B-cell lymphoma with *IRF4* rearrangement (included under follicular lymphoma)	Large B-cell lymphoma with *IRF4* rearrangement
*Burkitt-like lymphoma with 11q aberration*	*Large B-cell lymphoma with 11q aberration*	High-grade B-cell lymphoma with 11q aberration
Lymphomatoid granulomatosis	Lymphomatoid granulomatosis	Lymphomatoid granulomatosis
EBV + DLBCL, NOS	EBV + DLBCL, NOS	EBV + DLBCL
DLBCL associated with chronic inflammation (DLBCL-CI) Fibrin-associated diffuse large B-cell lymphoma	DLBCL-CI Fibrin-associated diffuse large B-cell lymphoma	DLBCL-CI
		Fibrin-associated large B-cell lymphoma
HHV8-negative effusion-based lymphoma (noted in differential diagnosis of PEL; not a distinct entity)	*HHV8 and EBV-negative primary effusion-based lymphoma*	Fluid overload-associated large B-cell lymphoma
Plasmablastic lymphoma (PBL)	PBL	PBL
Primary DLBCL of the CNS	Primary DLBCL of the CNS	Primary LBCL of immune-privileged sites (includes CNS, vitreoretinal, testis)
	Primary DLBCL of the testis	
Primary cutaneous diffuse large B-cell lymphoma, leg type	Primary cutaneous diffuse large B-cell lymphoma, leg type	Primary cutaneous diffuse large B-cell lymphoma, leg type
Intravascular large B-cell lymphoma	Intravascular large B-cell lymphoma	Intravascular large B-cell lymphoma
Primary mediastinal large B-cell lymphoma (PMBCL)	PMBCL	PMBCL
B-cell lymphoma, unclassifiable with features intermediate between DLBCL and classic HL	Mediastinal gray zone lymphoma	Mediastinal grey zone lymphoma
HGBCL, NOS	HGBCL, NOS	HGBCL, NOS -HGBCL with *MYC* and *BCL6* rearrangements
**Burkitt lymphoma** (BL)	BL	BL
**KSHV/HHV8-associated lymphoproliferative disorders and lymphomas**		
Primary effusion lymphoma (PEL) Subtype: extracavitary PEL	PEL Subtype: extra-cavitary PEL	PEL Subtype: extracavitary PEL
*HHV-8 positive DLBCL, NOS*	HHV-8 positive DLBCL, NOS	KSHV/HHV8 positive DLBCL
HHV-8 positive germinotropic lymphoproliferative disorder	HHV-8 positive germinotropic lymphoproliferative disorder	KSHV/HHV8 positive germinotropic lymphoproliferative disorder
	Multicentric Castleman Disease	KSHV/HHV8-associated multicentric Castleman Disease (included under Tumour-like lesions with B-cell predominance)
**Lymphoid proliferations and lymphomas associated with immune deficiency and dysregulation**		
Lymphoproliferative diseases associated with primary immune disorders Lymphomas associated with HIV infection Post-transplant lymphoproliferative disorders (PTLDs) Nondestructive (including plasmacytic hyperplasia PTLD, infectious mononucleosis (IM) PTLD, florid follicular hyperplasia (FFH) PTLD) Polymorphic PTLD Monomorphic PTLD (classified based on the lymphoma type to which they best correspond) Classic Hodgkin lymphoma PTLD Other iatrogenic immunodeficiency-associated LPDs	PTLDs Plasmacytic hyperplasia PTLD—IM PTLD FFH PTLD Polymorphic PTLD Monomorphic PTLD (B-, T/NK-cell types) Classic Hodgkin lymphoma PTLD Other iatrogenic immunodeficiency-associated LPDs	Hyperplasias arising in immune deficiency/dysregulation Polymorphic LPDs arising in immune deficiency/ dysregulation EBV + mucocutaneous ulcer Lymphomas arising in immune deficiency/dysregulation Inborn error of immunity-associated lymphoid proliferations and lymphoma *New nomenclature: 1) histologic diagnosis according to accepted criteria* *2) Presence or absence of oncogenic virus(es)* *3) Clinical setting/ immunodeficiency background*
*EBV* + *mucocutaneous ulcer (MCU)*	EBV + mucocutaneous ulcer	EBV + MCU (included in the category above)
	EBV + polymorphic B-cell LPD, NOS	Polymorphic LPDs arising in immune deficiency/ dysregulation (included above)

Italics—provisional entity

**Table 2:** Comparison of WHO-HAEM4R, ICC and WHO-HAEM5 Classification of Hodgkin Lymphoma

WHO HAEM4R	ICC	WHO HAEM5
*Hodgkin lymphoma*		
Nodular lymphocyte predominant Hodgkin lymphoma	Nodular lymphocyte predominant B-cell lymphoma (included among large B-cell lymphomas, not under Hodgkin lymphoma) -grade 1: Fan patterns A, B, C -grade 2: Fan patterns D, E & F	Nodular lymphocyte predominant Hodgkin lymphoma -Fan patterns are included, but grades are not assigned
Classic Hodgkin lymphoma -Nodular sclerosis classic Hodgkin lymphoma -Lymphocyte-rich classic Hodgkin lymphoma -Mixed-cellularity classic Hodgkin lymphoma -Lymphocyte-depleted classic Hodgkin lymphoma	Classic Hodgkin lymphoma -Nodular sclerosis classic Hodgkin lymphoma -Lymphocyte-rich classic Hodgkin lymphoma -Mixed-cellularity classic Hodgkin lymphoma -Lymphocyte-depleted classic Hodgkin lymphoma	Classic Hodgkin lymphoma -Nodular sclerosis classic Hodgkin lymphoma -Lymphocyte-rich classic Hodgkin lymphoma -Mixed-cellularity classic Hodgkin lymphoma -Lymphocyte-depleted classic Hodgkin lymphoma

**Table 3:** Comparison of WHO-HAEM4R, ICC and WHO-HAEM5 classification of plasma cell neoplasms and related disorders

WHO HAEM4R	ICC	WHO HAEM5
*Plasma cell neoplasms and related disorders*		
**Monoclonal gammopathies**		
Not included	Primary cold agglutinin disease	Cold agglutinin disease
IgM monoclonal gammopathy of uncertain significance (MGUS)	IgM MGUS -Lymphoplasmacytic lymphoma type -Plasma cell neoplasm type Non-IgM MGUS	IgM MGUS Non-IgM MGUS Monoclonal gammopathy of renal significance
**Plasma cell neoplasms**		
Plasmacytoma -Solitary plasmacytoma of bone -Extraosseous plasmacytoma	-Solitary plasmacytoma of bone -Extraosseous plasmacytoma	Plasmacytoma
Plasma cell myeloma Plasma cell myeloma variants -Smoldering plasma cell myeloma -Non-secretory myeloma -Plasma cell leukemia	Multiple myeloma (plasma cell myeloma) -Multiple myeloma NOS - Multiple myeloma with recurrent genetic abnormality - Multiple myeloma with *CCND* family translocation - Multiple myeloma with *MAF* family translocation - Multiple myeloma with *NSD2* family translocation - Multiple myeloma with hyperdiploidy	Plasma cell myeloma/multiple myeloma (PCM/MM) PCM/MM subtypes: -Smoldering plasma cell myeloma -Non-secretory myeloma -Plasma cell leukemia
Plasma cell neoplasms with associated paraneoplastic syndrome -POEMS syndrome -TEMPI syndrome (provisional)	Plasma cell neoplasms with associated paraneoplastic syndrome -POEMS syndrome -TEMPI syndrome	Plasma cell neoplasms with associated paraneoplastic syndrome -POEMS syndrome -TEMPI syndrome -AESOP syndrome
**Heavy chain diseases**		
Mu heavy chain disease Gamma heavy chain disease Alpha heavy chain disease	Mu heavy chain disease Gamma heavy chain disease Alpha heavy chain disease	Mu heavy chain disease Gamma heavy chain disease Alpha heavy chain disease
**Diseases with monoclonal immunoglobulin deposition**		
Primary amyloidosis	-Immunoglobulin light chain amyloidosis (AL) -Localized AL amyloidosis	Immunoglobulin-related amyloidosis (AL amyloidosis)
Light chain and heavy chain deposition diseases	Light chain and heavy chain deposition disease	Monoclonal immunoglobulin deposition disease

**Table 4:** Comparison of WHO-HAEM4R, ICC and WHO-HAEM5 classification of mature T-cell and NK-cell neoplasms and lymphoproliferative disorders

WHO-HAEM4R	ICC	WHO-HAEM5
*Mature T-cell and NK-cell neoplasms and lymphoproliferative disorders*		
**Mature T-cell and NK-cell leukemias**		
T-prolymphocytic leukemia	T-prolymphocytic leukemia	T prolymphocytic leukaemia
T-cell large granular lymphocytic leukemia	T-cell large granular lymphocytic leukemia	T large granular lymphocytic leukemia
*Chronic lymphoproliferative disorder of NK cells*	Chronic lymphoproliferative disorder of NK cells	NK-large granular lymphocytic leukemia
Adult T-cell leukemia/lymphoma	Adult T-cell leukemia/lymphoma	Adult T-cell leukemia/lymphoma
Sezary syndrome	Sezary syndrome	Sezary syndrome
Aggressive NK -cell leukemia	Aggressive NK-cell leukemia	Aggressive NK-cell leukemia
**Primary cutaneous T-cell lymphoid proliferations and lymphomas**		
*Primary cutaneous CD4-positive small/medium T-cell lymphoproliferative disorder*	Primary cutaneous CD4-positive small/medium T-cell lymphoproliferative disorder	Primary cutaneous CD4-positive small or medium T-cell lymphoproliferative disorder
*Primary cutaneous acral CD8-positive T-cell lymphoma*	Primary cutaneous acral CD8-positive T-cell lymphoproliferative disorder	Primary cutaneous acral CD8-positive T-cell lymphoproliferative disorder
Mycosis fungoides	Mycosis fungoides	Mycosis fungoides
Primary cutaneous CD30-positive lymphoproliferative disorders - Lymphomatoid papulosis - Primary cutaneous ALCL	Primary cutaneous CD30- positive T-cell lymphoproliferative disorders **-** Lymphomatoid papulosis - Primary cutaneous ALCL	Primary cutaneous CD30- positive T-cell lymphoproliferative disorders **-** Lymphomatoid papulosis - Primary cutaneous ALCL
Subcutaneous panniculitis-like T-cell lymphoma	Subcutaneous panniculitis-like T-cell lymphoma	Subcutaneous panniculitis-like T-cell lymphoma
*Primary cutaneous γδ T-cell lymphoma*	Primary cutaneous γδ T-cell lymphoma	Primary cutaneous γδ T-cell lymphoma
*Primary cutaneous CD8 positive aggressive epidermotropic cytotoxic T-cell lymphoma*	Primary cutaneous CD8 positive aggressive epidermotropic cytotoxic T-cell lymphoma	Primary cutaneous CD8 positive aggressive epidermotropic cytotoxic T-cell lymphoma
No counterpart (not distinguished from PTCL, NOS)	No counterpart (not distinguished from PTCL, NOS)	Primary cutaneous peripheral T-cell lymphoma, NOS
**Intestinal T-cell and NK-cell lymphoid proliferations and lymphomas**		
*Indolent T-cell lymphoproliferative disorder of the gastrointestinal tract*	Indolent clonal T-cell lymphoproliferative disorder of the gastrointestinal tract	Indolent T-cell lymphoma of the gastrointestinal tract
No counterpart	Indolent NK-cell lymphoproliferative disorder of the gastrointestinal tract	Indolent NK-cell lymphoproliferative disorder of the gastrointestinal tract
Enteropathy-associated T-cell lymphoma	Enteropathy-associated T-cell lymphoma	Enteropathy-associated T-cell lymphoma
Not listed, but discussed	Type II refractory celiac disease	Not listed, but discussed
Monomorphic epitheliotropic intestinal T-cell lymphoma	Monomorphic epitheliotropic intestinal T-cell lymphoma	Monomorphic epitheliotropic intestinal T-cell lymphoma
Intestinal T-cell lymphoma, NOS	Intestinal T-cell lymphoma, NOS	Intestinal T-cell lymphoma, NOS
Hepatosplenic T-cell lymphoma	Hepatosplenic T-cell lymphoma	Hepatosplenic T-cell lymphoma
**Anaplastic Large Cell Lymphoma (ALCL)**		
ALCL, ALK +	ALCL, ALK +	ALK + ALCL
ALCL, ALK-	ALCL, ALK-	ALK- ALCL
*Breast implant associated ALCL*	Breast implant-associated ALCL	Breast implant-associated ALCL
**T follicular helper cell lymphomas**		
Angioimmunoblastic T-cell lymphoma (AITL) and other nodal lymphomas of T follicular helper (TFH) cell origin -AITL -Follicular T-cell lymphoma -Nodal peripheral T-cell lymphoma with TFH phenotype	Follicular helper T-cell lymphoma (FhTCL) -FhTCL, AITL type (Angioimmunoblastic T-cell lymphoma) -FhTCL, Follicular type -FhTCL, NOS	Nodal T-follicular helper cell lymphoma (nTFHL) -nTFHL, AITL type -nTFHL, Follicular type -nTFHL, NOS
**Other Peripheral T-cell Lymphomas (PTCL)**		
PTCL, NOS	PTCL, NOS	PTCL, NOS
**EBV-Positive T-cell and NK cell Lymphomas**		
No counterpart	*Primary nodal EBV* + *T-cell/NK-cell lymphoma*	EBV-positive nodal T- and NK-cell lymphoma
Extranodal NK/T-cell lymphoma, nasal type	Extranodal NK/T-cell lymphoma, nasal type	Extranodal NK/T-cell lymphoma
**EBV-Positive T-cell and NK-cell Lymphoid Proliferations of Childhood**		
Severe mosquito bite allergy	Severe mosquito bite allergy	Severe mosquito bite allergy
Hydroa vaccinforme-like LPD	Hydroa-vacciniforme LPD -Classic -Systemic	Hydroa-vacciniforme LPD -Classic -Systemic
Chronic active EBV infection of T- and NK-cell type, systemic form	Chronic active EBV disease, systemic (T- and NK-cell phenotype)	Systemic chronic active EBV disease
Systemic EBV + T-cell lymphoma of childhood	Systemic EBV + T-cell lymphoma of childhood	Systemic EBV + T-cell lymphoma of childhood

Entities in italics: provisional

**Table 5:** Comparison of WHO-HAEM4R, ICC and WHO-HAEM5 classification of histiocytic/dendritic cell neoplasms

WHO HAEM4R	ICC	WHO HAEM5
*Histiocytic/dendritic cell neoplasms*		
**Langerhans cell neoplasms**		
Langerhans cell histiocytosis Langerhans cell sarcoma	Langerhans cell histiocytosis Langerhans cell sarcoma	Langerhans cell histiocytosis Langerhans cell sarcoma
**Other dendritic cell neoplasms**		
Indeterminate dendritic cell histiocytosis Interdigitating dendritic cell sarcoma	Indeterminate dendritic cell histiocytosis Interdigitating dendritic cell sarcoma	Indeterminate dendritic cell tumor Interdigitating dendritic cell sarcoma
**Histiocytic/macrophage neoplasms**		
Disseminated juvenile xanthogranuloma Erdheim-Chester disease Histiocytic sarcoma	Disseminated juvenile xanthogranuloma Erdheim-Chester disease Rosai-Dorfman-Destombes disease ALK-positive histiocytosis Histiocytic sarcoma	Juvenile xanthogranuloma Erdheim-Chester disease Rosai-Dorfman disease ALK-positive histiocytosis Histiocytic sarcoma
**Follicular dendritic cell neoplasms**		
Follicular dendritic cell sarcoma -Inflammatory pseudotumor-like follicular/fibroblastic dendritic cell sarcoma Fibroblastic reticular cell tumor	Follicular dendritic cell sarcoma EBV-positive inflammatory follicular dendritic cell sarcoma/fibroblastic reticular cell tumor Fibroblastic reticular cell sarcoma	Follicular dendritic cell sarcoma EBV-positive inflammatory follicular dendritic cell sarcoma Fibroblastic reticular cell tumor

### Chronic lymphocytic leukemia, small lymphocytic lymphoma, B-cell prolymphocytic leukemia and monoclonal B lymphocytosis

The approach to the diagnosis of monoclonal B lymphocytosis and chronic lymphocytic leukemia (CLL)/ small lymphocytic lymphoma (SLL) is similar in WHO-HAEM4R, WHO-HAEM5 and ICC, (1–3, 6–8) although there are some differences among the three. WHO-HAEM5 has eliminated “chronic lymphocytic leukemia/prolymphocytic leukemia”. Instead, WHO-HAEM5 defines *prolymphocytic progression* as representing greater than 15% prolymphocytes among lymphoid cells on a peripheral blood smear. The entity of *B-cell prolymphocytic leukemia* (B-PLL) has been eliminated in WHO-HAEM5, with the idea that cases diagnosed as B-PLL actually represent mantle cell lymphoma with leukemic involvement, chronic lymphocytic leukemia with prolymphocytic progression, or a primary splenic lymphoma with cytomorphology reminiscent of prolymphocytes (see below). The ICC requires > 55% prolymphocytes in the peripheral blood for a diagnosis of B-PLL (9) and restricts the diagnosis of B-PLL to cases in which transformed chronic lymphocytic leukemia, splenic marginal zone lymphoma, mantle cell lymphoma and hairy cell leukemia variant can be excluded.(2) Although the ICC does not explicitly define criteria for prolymphocytic transformation of CLL, it does state that “In addition to CLL, transformation to a neoplasm with prolymphocytic features has been described in rare cases of splenic marginal zone lymphomas”, indicating that the ICC does indeed recognize prolymphocytic transformation of CLL.(9).

High-grade transformation of CLL (Richter transformation, RT) usually takes the form of diffuse large B-cell lymphoma (DLBCL), while a minority of RT have features of Hodgkin lymphoma.(10–12) ICC describes two types of Hodgkin lymphoma-like Richter transformation of CLL: type 1, in which Hodgkin-Reed-Sternberg (HRS) cells are present in a background of CLL/SLL, and type 2, in which HRS cells are found in the mixed inflammatory background typical of sporadic classic Hodgkin lymphoma.(9, 13) Optimal management of such cases is not yet defined.(14) As the concept of HRS cells without a Hodgkin-like background infiltrate as transformation is considered preliminary, WHO-HAEM5 continues to require a non-neoplastic microenvironment similar to that in sporadic classic Hodgkin lymphoma to establish a diagnosis of Hodgkin lymphoma-like Richter transformation.(7) Of note, some cases of DLBCL or CHL occurring in CLL patients status post chemotherapy, particularly if they are EBV + , may not be clonally related to the CLL, and may be better considered as a type of lymphoma associated with immune deficiency and dysregulation. (7).

### Splenic B-cell lymphomas and leukemias

Classification of hairy cell leukemia and splenic marginal cell lymphoma is essentially unchanged in the WHO-HAEM5 and ICC, compared to WHO-HAEM4R, except for updated information regarding pathologic features of these B-cell neoplasms.(2, 15, 16) The WHO-HAEM4R entity of *splenic B cell lymphoma/leukemia, unclassifiable*, which included splenic diffuse red pulp small B-cell lymphoma and hairy cell leukemia variant,(17) is unchanged in ICC.(2) That category is removed in WHO-HAEM5. Splenic diffuse red pulp small B-cell lymphoma, a provisional entity in WHO-HAEM4R and ICC, is accepted as an entity in WHO-HAEM5. Hairy cell leukemia variant is also a provisional entity in WHO-HAEM4R and ICC but that diagnostic term is eliminated in WHO-HAEM5, because of the lack of a clear biological relationship between hairy cell leukemia and cases classified as hairy cell leukemia variant. WHO-HAEM5 introduced the term *splenic B-cell lymphoma/leukemia with prominent nucleoli* to encompass most cases that would otherwise be designated hairy cell leukemia variant as well as cases of CD5-negative prolymphocytic leukemia.(18) The diagnosis of splenic B-cell lymphoma/leukemia with prominent nucleoli can be made in patients known to have splenomegaly, with circulating B-lineage neoplastic cells with the appropriate cytomorphology. A splenectomy specimen is not mandatory to establish this diagnosis. Splenic B-cell lymphoma/leukemia with prominent nucleoli is a placeholder category that may also include cases of some low-grade splenic B cell lymphomas, presenting at the time of histologic progression. Thus, this is likely a heterogeneous category requiring additional study.

### Lymphoplasmacytic lymphoma and IgM monoclonal gammopathy of uncertain significance (IgM MGUS)

Lymphoplasmacytic lymphoma is a low-grade B-cell lymphoma with plasmacytic differentiation, almost always IgM + (rarely IgA + or IgG +) and associated with bone marrow involvement, typically associated with an IgM M component, and almost always with an underlying *MYD88* p.L265P. In accordance with the results of the Second International Workshop on Waldenström’s macroglobulinemia,(19) a diagnosis of lymphoplasmacytic lymphoma can be established in cases with abnormal lymphoplasmacytic aggregates in the marrow even when they comprise less than 10% of the total marrow cellularity, in the appropriate clinical context, in ICC.(2) WHO-HAEM5 requires significant bone marrow infiltration but does not stipulate a specific threshold.(20).

A diagnosis of IgM MGUS requires a serum monoclonal protein of less than 3 g/dL, fewer than 10% clonal lymphoplasmacytic cells in the bone marrow, and absence of anemia, constitutional symptoms, hyperviscosity, lymphadenopathy or organomegaly that can be attributed to the lymphoproliferative disorder,(21) as well as absence of lymphoid aggregates that would be sufficient for a diagnosis of lymphoplasmacytic lymphoma.(2) ICC denotes two types of IgM MGUS: Lymphoplasmacytic lymphoma type (representing a precursor of lymphoplasmacytic lymphoma) and a much less common plasma cell type (representing a precursor of plasma cell myeloma/multiple myeloma).

*Cold agglutinin disease* (CAD; WHO-HAEM5),(22) designated *primary cold agglutinin disease* in ICC,(2) is a newly introduced category, separate from lymphoplasmacytic lymphoma and IgM MGUS, representing an autoimmune hemolytic anemia mediated by monoclonal cold agglutinins, related to an underlying clonal B-cell proliferation, in the absence of infection or overt lymphoma. CAD must be distinguished from early marrow involvement by lymphoplasmacytic lymphoma. There is no association with *MYD88* p.L265P.(22).

### Marginal zone lymphomas

In WHO-HAEM4R, *primary cutaneous marginal zone lymphoma* was not separated as a distinct entity from other extranodal marginal zone lymphomas; in ICC and WHO-HAEM5,(23) it is included as a distinct entity. Of note, in ICC, the term *primary cutaneous marginal zone lymphoproliferative disorder* is used, given the generally indolent behavior of these neoplasms, while WHO-HAEM5 retains a designation of primary cutaneous marginal zone lymphoma, while fully acknowledging the indolent behavior of this disease.(2).

In WHO-HAEM4R, *pediatric nodal marginal zone lymphoma* (PNMZL) is discussed within the chapter on *nodal marginal zone lymphoma* (NMZL),(24) and ICC retains this approach, including PNMZL under the heading of NMZL(2) while WHO-HAEM5 includes pediatric nodal marginal zone lymphoma as a distinct entity.(25).

### Follicular lymphoma

WHO-HAEM4R mandated grading of follicular lymphoma (FL) as grades 1-3A and 3B, and included *testicular follicular lymphoma*, in situ* follicular neoplasia*, and *duodenal-type FL* under the heading of FL. *Pediatric-type FL* and *primary cutaneous follicle center lymphoma* were included as distinct entities.(26) WHO-HAEM4R also noted the occurrence of rare, predominantly diffuse follicle center lymphomas, often presenting with inguinal lymphadenopathy, negative for BCL2 rearrangement, expressing CD23 and harboring loss of 1p36. ICC maintained these categories and formally added *BCL2-rearrangement negative, CD23* + *follicle center lymphoma* (largely corresponding to the predominantly diffuse follicle center lymphomas noted in WHO-HAEM4R) and *testicular follicular lymphoma* as distinct entities.(2).

WHO-HAEM5 took a different approach, renaming follicular lymphomas with a mixture of centrocytes and centroblasts as *classic follicular lymphoma* (cFL), and making grading optional. In contrast to WHO-HAEM4R, the diffuse areas of cFL with sufficient centroblasts to fulfill criteria for FL, grade 3A are not automatically designated DLBCL. A diffuse lymphoma with 15 centroblasts/hpf is still composed mainly of centrocytes, and by conventional criteria would not fulfill criteria for a diagnosis of DLBCL. The optimal biological cut-off regarding number or proportion of large cells for a diagnosis of a component of DLBCL in this setting requires additional study. FL composed entirely of centroblasts (FL, grade 3B) was renamed *follicular large B-cell lymphoma*.(27) WHO-HAEM5 also included a subcategory of *FL with unusual cytologic features*, including cases with blastoid chromatin, and those composed of large centrocytes and a subcategory of *FL with a predominantly diffuse growth pattern*,(27) overlapping with the ICC category of BCL2-rearrangement negative, CD23 + follicle center lymphoma. Rather than in situ follicular neoplasia, WHO-HAEM5 introduced the term *in situ follicular B-cell neoplasm*. Like WHO-HAEM4R and ICC, WHO-HAEM5 includes duodenal-type FL, pediatric-type FL and primary cutaneous follicle center lymphoma as entities, but does not include testicular lymphoma as a distinct entity because of its extreme rarity and some features overlapping with pediatric-type FL.

### Mantle cell lymphoma

There are a few minor changes in nomenclature from WHO-HAEM4R(28) to WHO-HAEM5 and ICC pertaining to mantle cell lymphoma (MCL). Under MCL in WHO-HAEM4R, there were categories of in situ MCL and non-nodal MCL, both with a better prognosis than other cases of MCL. In ICC, the term *in situ MCL* is retained and non-nodal MCL becomes *leukemic non-nodal MCL*.(2) Corresponding terms in WHO-HAEM5 are *in situ mantle cell neoplasm* and *leukemic non-nodal MCL*.(29–31).

### Large B-cell lymphomas

Under the category of *diffuse large B-cell lymphoma (DLBCL), NOS*, ICC and WHO-HAEM5 formally recognize germinal center B-cell and activated B-cell types. In addition, WHO-HAEM5 recognizes morphologic subtypes: centroblastic, immunoblastic, and anaplastic. DLBCL with *MYC* and *BCL6* rearrangements is currently included as a genetic subtype under DLBCL, NOS, in WHO-HAEM5,(32) in contrast to WHO-HAEM4R(33) and ICC.(2).

In WHO-HAEM4R, the category of *high grade B-cell lymphoma (HGBCL) with MYC and BCL2 and/or BCL6 rearrangements* included all diffuse B-cell lymphomas with either large cell or high-grade cytomorphology and a *MYC* rearrangement accompanied by rearrangement of *BCL2* or *BCL6* or both *BCL2* and *BCL6*.(34) ICC maintains a category of HGBCL with *MYC* and *BCL2* rearrangement, and includes HGBCL with *MYC* and *BCL6* rearrangements as a provisional category, pending accumulation of additional data on this type of lymphoma.(2) In WHO-HAEM5, lymphomas with concurrent *MYC* and *BCL2* rearrangements are classified as either DLBCL or as HGBCL (based on cytomorphology) with *MYC* and *BCL2* rearrangement.(35) Cases with DLBCL morphology and concurrent *MYC* and *BCL6* rearrangement (without *BCL2* rearrangement) are not included as HGBCLs but rather under DLBCL, NOS.

Diffuse B-cell lymphomas with concurrent MYC and BCL2 rearrangements are aggressive neoplasms, although there is some pathologic heterogeneity. As noted above, the lymphomas can have large cell or “high-grade” morphology. They usually have a GCB immunophenotype with expression of BCL2. At the genetic level, many cases have a *MYC* rearrangement with an immunoglobulin heavy chain or less often, light chain partner. In such cases, MYC expression is uniformly high.(36) In other cases, *MYC* has a non-immunoglobulin (Ig) partner, and in these cases, MYC expression is variable. Among the non-Ig partners is *BCL6*; in one study, the majority of lymphomas with rearrangements of *MYC*, *BCL2* and *BCL6* (“triple-hit” lymphomas) harbored *BCL6::MYC* rearrangements,(36) suggesting that such cases may be considered pseudo-triple hit lymphomas. *MYC* transactivation may not occur in cases in which *MYC* has a non-Ig partner,(36) suggesting the possibility of less aggressive behavior.

Rare follicular lymphomas have concurrent *MYC* and *BCL2* rearrangements.(37–39) These follicular lymphomas may on average be of higher histologic grade and have somewhat more aggressive behavior than unselected follicular lymphomas, but they are less aggressive than HGBCL or DLBCL with concurrent *MYC* and *BCL2* rearrangement and are not included in this diagnostic category in WHO-HAEM4R, WHO-HAEM5, or ICC.

WHO-HAEM4R included *large B-cell lymphoma with IRF4 rearrangement*, a de novo B-cell lymphoma with a follicular and/or diffuse pattern, strong expression of IRF4/MUM1 and a favorable prognosis, as a provisional entity. ICC and WHO-HAEM5 recognize large B-cell lymphoma with IRF4 rearrangement (LBCL-IRF4) as a distinct entity. However, in the ICC, LBCL-IRF4 are listed under follicular lymphomas, while in WHO-HAEM5, they are under large B-cell lymphomas.(2, 40).

WHO-HAEM4R introduced a category of *Burkitt-like lymphoma with 11q aberration*, an aggressive mature B-cell lymphoma with a characteristic chromosome 11q-gain/loss pattern. *MYC* rearrangement excludes the diagnosis. Because some cases have large cell morphology and have genetic features distinct from those of Burkitt lymphoma and more akin to DLBCL of germinal center B-cell subtype, ICC modified the original nomenclature to *large B-cell lymphoma with 11q aberration*,(2) while in WHO-HAEM5, nomenclature was modified to *high-grade B-cell lymphoma with 11q aberration*, since most cases have high-grade histologic features, potentially mimicking Burkitt lymphoma.(41).

EBV + DLBCL, NOS is a large B-cell lymphoma in which the vast majority of neoplastic cells harbor EBV. By definition, patients do not have a history of lymphoma or specific immunodeficiency (other than immunosenescence), and criteria for other EBV + lymphoproliferative disorders or lymphomas are not fulfilled.(42) ICC retained the name of the entity, *EBV* + *DLBCL, NOS* used in WHO-HAEM4R,(2) while *EBV* + *DLBCL*, without the “NOS” was adopted in WHO-HAEM5.(43) While there are no major differences for the diagnosis of this lymphoma among the three classifications, ICC requires > 80% of neoplastic cells be EBV + , while WHO-HAEM5 requires only that the majority of tumor cells are EBV + .

WHO-HAEM4R included a category of *DLBCL with chronic inflammation* (DLBCL-CI), an EBV + DLBCL arising in a closed anatomic space, associated with prolonged chronic suppurative inflammation, the prototype of which is *pyothorax-associated lymphoma*.(44–46) Fibrin-associated DLBCL, a large B-cell lymphoma, almost always EBV + , typically presenting as a non-mass forming, incidental finding in an anatomically isolated space (e.g., within cysts, pseudocysts, stroma of atrial myxomas, blood clots, etc.) was classified as a subtype of DLBCL-CI in WHO-HAEM4R.(44) ICC retained this nomenclature.(2) DLBCL-CI is included in WHO-HAEM5,(47) but, in contrast to WHO-HAEM4R and ICC, *fibrin-associated large B-cell lymphoma* (FA-LBCL) is classified as an entity distinct from DLBCL-CI.(1, 48) DLBCL-CI typically behaves in an aggressive manner while FA-LBCL is associated with an excellent prognosis if completely excised.(44–48).

ICC introduced a category of *EBV* + *polymorphic B-cell lymphoproliferative disorder, NOS*, for EBV + B-cell proliferations, arising in patients with or without known immunodeficiency, that do not fulfill criteria for lymphoma, whether due to a polymorphous composition or a small, suboptimal biopsy.(2, 3) In patients with known immunodeficiency, such cases would most closely correspond to a polymorphic lymphoproliferative disorders arising in immune deficiency/ dysregulation in WHO-HAEM5, (49) but there is no specific equivalent in WHO-HAEM5 in immunocompetent patients.

The term *HHV8-negative effusion-based lymphoma* was used in WHO-HAEM4R in the context of the differential diagnosis of primary effusion lymphoma but was not included as a distinct entity. (50) This type of lymphoma is a large B-cell lymphoma, presenting as an effusion involving one or more body cavities without a solid component, but that is HHV8-negative, in contrast to primary effusion lymphoma.(45, 46, 50) Remarkably, some patients with these lymphomas achieve a complete remission following drainage of the effusion alone.(46, 51, 52) ICC adopted a similar name for this type of lymphoma, *HHV8- and EBV-negative primary effusion based lymphoma,* requiring that they be negative for EBV as well as for HHV8.(2) EBV + cases in this category may be associated with an inferior prognosis and often occur in individuals with an underlying immunodeficiency,(45, 46, 51) and were thus excluded by the ICC in order to have a more uniform diagnostic entity. These lymphomas typically occur in older patients with medical conditions associated with fluid overload. In WHO-HAEM5, the name *fluid overload-associated large B-cell lymphoma* was given to this type of lymphoma, with the idea of emphasizing its positive, rather than its negative attributes.(1, 53) EBV + cases are not excluded, but if EBV is present, that finding should be indicated in the diagnosis, and if an immunodeficiency state is known (other than immunosenescence), the case be diagnosed according to the rules for lymphomas/lymphoproliferations arising in immune deficiency and dysregulation (see below).

WHO-HAEM4R and ICC both include *primary DLBCL of the central nervous system* (CNS) as an entity, and ICC also recognizes primary DLBCL of the testis as an entity, because of its distinctive features.(2) WHO-HAEM5 recognizes a category of *primary large B-cell lymphoma of immune-privileged sites* (CNS, vitreoretinal and testicular primary sites). (54) These lymphomas are characterized by genetic changes that lead to immune escape, often have concurrent *MYD88* and *CD79B* mutations, and share the C5/MCD/MYD88 genomic signature.(54) The ICC considered adopting a similar category of extranodal lymphoma, ABC (non-GCB) type for selected primary extranodal lymphomas, but ultimately did not.(2) Both ICC and WHO-HAEM5 note that certain other lymphomas, such as primary cutaneous DLBCL, leg-type and primary DLBCL of the breast and ovary, share features with primary DLBCL of the CNS and testis,(2, 54) and may eventually be considered to represent primary large B-cell lymphomas of immune-privileged sites.(54).

The WHO-HAEM4R entity, B-cell lymphoma, unclassifiable, with features intermediate between DLBCL and classic Hodgkin lymphoma, has long been informally designated as “gray zone lymphoma.” Both ICC and WHO-HAEM5 have adopted the designation *mediastinal gray zone lymphoma* (MGZL) for this unusual type of lymphoma.(2, 55) WHO-HAEM5 divides MGZL into those with a classic Hodgkin lymphoma (CHL)-like histology and those with primary mediastinal large B-cell lymphoma (PMBL)-like histology. Per WHO-HAEM5, CHL-like MGZL shows confluent growth of pleomorphic cells within a variably abundant microenvironment and dense fibrotic stroma; uniform strong expression of CD20, PAX5, and at least one additional B-cell marker (CD19, CD79a, BOB1, OCT2); and positive expression of CD30. PMBL-like MGZL shows monomorphic sheets of medium-sized to large neoplastic cells within a variably dense fibrotic stroma; strong and uniform positive expression of CD30 and partial or complete loss of B-cell markers, or strong CD15 expression.(55) Of note, ICC excludes EBV + lymphomas from this category, while WHO-HAEM5 states that EBV + MGZL is extremely rare, and recommends that other diagnoses be seriously considered if tumor cells are EBV + .

A number of large B-cell lymphomas have uniform nomenclature in WHO-HAEM4R, ICC and WHO-HAEM5. These include: *T-cell/histiocyte-rich large B-cell lymphoma*, a large B-cell lymphoma with a microenvironment consisting of abundant T cells and histiocytes;(56) *ALK* + *large B-cell lymphoma*, an aggressive large B-cell lymphoma with a poor prognosis, characterized by ALK protein expression and a translocation involving *ALK* and one of various partners, the most common of which is *CLTC* which encodes clathrin;(57) *plasmablastic lymphoma*, a large B-cell lymphoma with a plasma cell phenotype which is often EBV + and often occurs in the setting of immunodeficiency;(58) *primary cutaneous diffuse large B-cell lymphoma, leg-type*, a large B-cell lymphoma with a non-GCB immunophenotype, that is the most aggressive of the primary cutaneous B-cell lymphomas;(59) *intravascular large B-cell lymphoma*, a lymphoma that is entirely or almost entirely, confined to the lumens of blood vessels;(60) and primary mediastinal large B-cell lymphoma (PMBL), a distinctive lymphoma of putative thymic B-cell origin. Large B-cell lymphomas with features reminiscent of PMBL, but arising outside the mediastinum, are generally better considered to represent DLBCL, NOS. (61) *Lymphomatoid granulomatosis* (LYG), an EBV + angiocentric, angiodestructive lymphoproliferative disorder of large B cells involving the lungs and sometimes other extranodal sites, *high-grade B-cell lymphoma (HGBCL), NOS* and *Burkitt lymphoma* also share uniform nomenclature in WHO-HAEM4R, ICC and WHO-HAEM5. Of note, ICC and WHO-HAEM5 both require absence of a specific underlying immunodeficiency for a diagnosis of LYG, in contrast to WHO-HAEM4R. The term HGBCL, NOS is used for histologically high-grade B-cell lymphomas without specific underlying genetic abnormalities that would mandate classification as another entity, such as concurrent rearrangements of *BCL2* and *MYC*.(2) WHO-HAEM5 emphasizes the importance of the presence or absence of Epstein-Barr virus (EBV) in cases of Burkitt lymphoma which may correlate more precisely with pathogenesis and genetic features than the traditional epidemiological division into endemic, sporadic and immunodeficiency-associated Burkitt lymphoma.(62).

### KSHV/HHV8-associated B-cell lymphoid proliferations and lymphomas

The KSHV/HHV8-associated lymphoproliferative disorders share similar nomenclature in WHO-HAEM4R, ICC and WHO-HAEM5.(1–3) They include *primary effusion lymphoma* (PEL); *KSHV/HHV8* + *DLBCL* in WHO-HAEM5 and *HHV8* + *DLBCL, NOS* in ICC; *KSHV/HHV8* + *germinotropic lymphoproliferative disorder* in WHO-HAEM5 and *HHV8* + *germinotropic lymphoproliferative disorder* (GLPD) in ICC; and *KSHV/HHV8-associated multicentric Castleman disease* (included in the section on Tumour-like lesions with B-cell predominance in WHO-HAEM5) and multicentric Castleman disease in ICC.

PEL is a rare, aggressive HHV8 + large B-cell lymphoma arising mainly in young to middle-aged HIV + males or in older adults with the immunosenescence of aging or with another source of immunosuppression. PEL almost always fails to express pan-B-cell antigens and often harbors EBV, especially in HIV + patients.(45, 46, 63–65) Extracavitary PEL is a subtype of PEL, presenting as a mass lesion, often in an extranodal site. KSHV/HHV8 + DLBCL is a large B-cell lymphoma typically arising in immunocompromised patients and mainly involving lymph nodes and spleen. Neoplastic cells are typically IgM + and negative for EBV.(66) KSHV/HHV8 + GLPD is a rare lymphoproliferative disorder, mainly affecting older adults without a specific underlying immunodeficiency, in which HHV8 + large B cells colonize the germinal centers of lymphoid follicles. The HHV8 + cells are usually co-infected by EBV. The course is indolent in most cases.(67–70) KSHV/HHV8 + MCD is an uncommon lymphoproliferative disorder typically characterized by HHV8 + , IgM lambda + plasmablasts, which are usually scattered in the mantles of lymphoid follicles of lymph nodes with histologic features similar to those of idiopathic multicentric Castleman disease, and accompanied by systemic symptoms related to proinflammatory hypercytokinemia. (71–73).

### Lymphoid proliferations and lymphomas associated with immune deficiency and dysregulation

The classification of post-transplant lymphoproliferative disorders (PTLDs) is essentially unchanged from WHO-HAEM4R to ICC. In WHO-HAEM5, a different approach to classification of *lymphoid proliferations and lymphomas associated with immune deficiency and dysregulation* was adopted. Following on the recommendations of the Society for Hematopathology/ European Association for Hematopathology 2015 workshop, a three-part diagnosis was recommended: 1. Histologic diagnosis; 2. Presence or absence of oncogenic virus; 3. Type of immunodeficiency. Thus, a lesion previously classified as an EBV + monomorphic PTLD, consistent with DLBCL, would be diagnosed using WHO-HAEM5 nomenclature as DLBCL, EBV + , post-transplant. This nomenclature is broadly applicable to many different clinical settings and types of immunodeficiency.(74) WHO-HAEM4R and ICC have a separate category of *other iatrogenic immunodeficiency-associated lymphoproliferative disorders* for classification of such disorders outside the transplant setting.(2).

WHO-HAEM5 also includes *EBV* + *mucocutaneous ulcer* (EBV-MCU)(75) and *inborn error of immunity-associated lymphoid proliferations and lymphomas*(76) under the heading of lymphoid proliferations and lymphomas associated with immune deficiency and dysregulation. EBV-MCU was included in WHO-HAEM4R as a provisional entity, and is included in ICC, but separate from the immunodeficiency-associated lymphoproliferative disorders.(2) WHO-HAEM5 and ICC agree that EBV-MCU typically presents as a single ulcer; however, WHO-HAEM5 does not exclude a diagnosis of EBV-MCU in cases with multiple lesions within a single anatomic site that otherwise fulfill criteria for EBV-MCU, while more than one lesion would lead to a diagnosis of a different type of EBV + lymphoproliferative disorder or lymphoma, per the ICC.

## Hodgkin lymphoma

In WHO-HAEM5, the section on Hodgkin lymphoma is located between sections on Mature B-cell neoplasms and Plasma cell neoplasms, emphasizing the established B lineage of the neoplastic cells, while in WHO-HAEM4R, Hodgkin lymphoma is in the section that follows T-cell lymphomas. The classification of Hodgkin lymphoma used in WHO-HAEM4R(77) is maintained in WHO-HAEM5. ICC maintains the same classification of classic Hodgkin lymphoma but has changed the name of *nodular lymphocyte predominant Hodgkin lymphoma* (NLPHL), used in WHO-HAEM4R and WHO-HAEM5, to *nodular lymphocyte predominant B-cell lymphoma* (NLPBL), and has moved this entity from under the heading of Hodgkin lymphoma to be included among the large B-cell lymphomas (Table 2).(2) This nomenclature has the advantage of emphasizing the B lineage of this lymphoma and its risk of progression to DLBCL or a lymphoma with features of T-cell/histiocyte-rich large B-cell lymphoma, although the disadvantage of giving a somewhat non-specific description, as several types of lymphoma have a nodular pattern and are rich in lymphocytes. Retaining “Hodgkin” in the name in WHO-HAEM5 concisely indicates that the neoplastic cell is a large B cell present in relatively small numbers in an abundant microenvironment, while “lymphocyte predominant” describes the microenvironment.

ICC has also introduced grading of NLPBL. Variant patterns as described by Fan et al.(78) that have been associated with an inferior outcome include those with neoplastic cells outside B-cell rich nodules and those with decreased B cells in the microenvironment,(79) and these are assigned a grade of 2, while Fan patterns with retention of the B-cell rich microenvironment are designated grade 1.(2).

## Plasma cell neoplasms and related disorders

Classification of plasma cell neoplasms is similar in WHO-HAEM4R, ICC and WHO-HAEM5 (Table 3).(2, 80) ICC recognizes multiple myeloma with recurrent genetic abnormalities as distinct entities; these genetic changes are noted in WHO-HAEM4R and WHO-HAEM5 without designating them as distinct entities. Heavy chain diseases (mu, gamma and alpha) and diseases associated with immunoglobulin deposition (immunoglobulin light chain amyloidosis and monoclonal immunoglobulin deposition disease) are included in both ICC and WHO-HAEM5, with similar nomenclature. Like WHO-HAEM4R, ICC uses the term *light chain and heavy chain deposition disease*,(2) while WHO-HAEM5 has adopted *monoclonal immunoglobulin deposition disease*.(81).

## Mature T-cell and NK-cell Leukemias

The mature lymphoid T and NK-cell leukemias are well defined and very little substantive differences exist between the ICC and WHO-HAEM5 (Table 4). The WHO-HAEM4R name *T-cell large granular lymphocytic leukemia* (T-LGLL) was carried forward in ICC while WHO-HAEM5 revised the name to *T-large granular lymphocytic leukemia*. *Chronic lymphoproliferative disorder of NK cells* from the WHO-HAEM4R is retained in the ICC but elevated to a definitive entity from provisional entity. However, the WHO-HAEM5 commits to the term *NK-large granular lymphocytic leukemia*, reflecting the monoclonal nature of the disease and similarities to T-LGLL.

### Primary Cutaneous T-cell Lymphoid Proliferations and Lymphomas

As shown in Table 4, two minor differences exist for the primary cutaneous T-cell proliferations and lymphomas. While the WHO-HAEM4R terminology of *primary cutaneous CD4-positive small/medium T-cell LPD* is retained and elevated to definitive entity in ICC, WHO-HAEM5 emphasizes the acceptance of cytologic variability in the lesions, consisting of small or medium sized lymphocytes. Unlike the ICC, the WHO-HAEM5 specifically calls out *primary cutaneous peripheral T-cell lymphoma, not otherwise specified* (pcPTCL, NOS) for the very uncommon cases that do not fit other defined categories, while in the ICC the qualifier “primary cutaneous” is not recognized and cases designated simply as PTCL, NOS. Under the WHO-HAEM5, cases of primary cutaneous PTCL, NOS (pcPTCL, NOS) with 2 or more Tfh markers would still be designated as pcPTCL, NOS but qualified as having a Tfh phenotype (ie. PC PTCL, NOS with a Tfh phenotype). *Primary cutaneous acral CD8* + *T-cell lymphoma* in the WHO-HAEM4R is designated as a lymphoproliferative disorder, rather than lymphoma as well as elevated to a definitive entity status in both the WHO-HAEM5 and ICC.

### Intestinal T-cell and NK-cell lymphoid proliferations and lymphomas and hepatosplenic T-cell lymphoma

Minor differences exist in the ICC and WHO-HAEM5 for this group of lymphoid proliferations. *Enteropathy-associated T-cell lymphoma* (EATL), a well described entity, retains the same name in both systems. However, since *refractory celiac disease type II* (RCD II) shows intraepithelial lymphocytes that often have an abnormal TCR-silent phenotype, typically are monoclonal, usually harbor somatic mutations in JAK/STAT pathway genes, and have a high risk for lymphoma development, the ICC includes RCD II in the classification under EATL.(82–84) *Monomorphic epitheliotropic intestinal T-cell lymphoma* and *intestinal T-cell lymphoma, NOS* remain unchanged in both the ICC and WHO-HAEM5. While the rare *indolent T-cell LPD of the GI tract* from the WHO-HAEM4R is retained in the ICC with addition of “clonal” as a qualifier, the WHO-HAEM5 preferred the term *indolent T-cell lymphoma of the GI tract* based on the fact that these proliferations are monoclonal, harbor somatic mutations, are often highly symptomatic and may uncommonly evolve to aggressive disease.(85–88) Both systems recognize as an entity an NK-cell counterpart previously known as NK-cell enteropathy or lymphomatoid gastropathy.(89–91) Both rename this as *indolent NK cell lymphoproliferative disorder of the gastrointestinal tract*. Finally, *hepatosplenic lymphoma* is unchanged from the WHO-HAEM4R in both new systems.

### Anaplastic large cell lymphoma (ALCL)

The WHO-HAEM4R terminology *ALCL, ALK* + *and ALK-* is retained in the ICC; however, the WHO-HAEM5 leads with ALK status (ALK + ALCL and ALK- ALCL). *Breast implant-associated ALCL* is elevated to a defined entity from provisional status and terminology is identical in the ICC and WHO-HAEM5.

### Nodal T follicular helper cell lymphomas

These lymphomas are recognized in the WHO-HAEM4R, ICC and WHO-HAEM5. In WHO-HAEM5, they are termed *Nodal T-follicular helper cell lymphomas*, whereas in the ICC the group is termed *Follicular helper T-cell lymphomas*. Both WHO-HAEM5 and ICC recognize the same three subtypes: angioimmunoblastic type, follicular type and an NOS category for nodal PTCLs that are found to have a T follicular helper (Tfh) phenotype as defined by 2 or more Tfh markers (such as PD1, ICOS, CXCL13, CD10, BCL6, CXCR5), but without the distinctive features of the other two types.

### Peripheral T-cell lymphoma (PTCL) NOS

These remain a diagnosis of exclusion for nodal and extranodal PTCLs that do fit into other categories.

### EBV-positive T-cell and NK-cell lymphomas

WHO-HAEM5 and ICC both introduced a category of EBV-positive nodal T- and NK-cell lymphoma (WHO-HAEM5)/Primary nodal EBV + T-cell/NK-cell lymphoma (ICC) to recognize that uncommon nodal T- and NK-cell lymphomas occur that are EBV + .(3, 92, 93) The WHO-HAEM4R entity of extranodal NK/T-cell lymphoma, nasal-type, is maintained in ICC.(88) In WHO-HAEM5, the name is shortened to extranodal NK/T-cell lymphoma.(94).

### EBV-positive T-cell and NK-cell lymphoid proliferations and lymphomas of childhood

*Severe mosquito bite allergy*, a rare systemic EBV-associated reaction to mosquito bite in Asia, Mexico, and Central/South America was part of the WHO-HAEM4R and is retained in the ICC and WHO-HAEM5. *Hydroa vacciniforme-like LPD* in the WHO-HAEM4R is now considered *Hydroa vaccinforme LPD* with a classic form demonstrating limited sun-exposed skin involvement and a systemic form, often with non-sun-exposed skin and multiorgan involvement.(95) Terminology is identical in the ICC and WHO-HAEM5. *Chronic active EBV infection of T and NK cell type, systemic form* in the WHO-HAEM-4R is updated to *systemic chronic active EBV disease* in the WHO-HAEM5. The ICC also used “disease” to describe the entity and retains similar terminology and explicit lineage types (*Chronic active EBV disease, systemic (T- and NK-cell phenotype)*). The most aggressive disease in this category, *systemic EBV* + *T-cell lymphoma of childhood*, is identical in both new systems.

## Histiocytic/dendritic cell neoplasms

WHO-HAEM4R, ICC and WHO-HAEM5 all include histiocytic and dendritic cell neoplasms, with similar or identical nomenclature, although Rosai Dorfman Disease and ALK + histiocytosis, neither of which were included in WHO-HAEM4R, are included in both ICC and WHO-HAEM5 (Table 5). (2, 96–98) WHO-HAEM5 also includes a myofibroblastic tumour (*intranodal palisaded myofibroblastoma*) and spleen-specific vascular-stromal tumours (*littoral cell angioma*, *splenic hamartoma* and *sclerosing angiomatoid nodular transformation of spleen*).

## Summary

A vast amount of literature has been published regarding the classification of hematolymphoid neoplasms in the past several years, and this review attempts to highlight the most important differences among WHO-HAEM4R, ICC and WHO-HAEM5. While there are differences in the classification of mature lymphoid neoplasms between ICC and WHO-HAEM5, they are mostly relatively minor and familiarity with both classifications will lead to use of the correct nomenclature. Including nomenclature from both ICC and WHO-HAEM5, when it differs, is recommended for use in diagnostic pathology reports. The goal of future efforts is to return to a single classification system, incorporating input from a Clinical Advisory Committee into the next edition of the WHO Classification of Haematolymphoid Neoplasms, WHO-HAEM6.

## Data Availability

Making original data available is not applicable for this review.
