# Correction: Mature B, T and NK-cell, plasma cell and histiocytic/dendritic cell neoplasms: classification according to the World Health Organization and International Consensus Classification

**DOI:** 10.1186/s13045-024-01585-y

**Published:** 2024-08-29

**Authors:** Judith A. Ferry, Brian Hill, Eric D. Hsi

**Affiliations:** 1 Department of Pathology, Massachusetts General Hospital, Harvard Medical School, 55 Fruit Street, Boston, MA 02114 USA; 2 Department of Hematology and Medical Oncology, Cleveland Clinic, Cleveland, OH USA; 3 Department of Laboratory Medicine and Pathology, Mayo Clinic, Rochester, MN USA

**Journal of Hematology & Oncology (2024) 17:51**

10.1186/s13045-024-01570-5

The original article contains typesetting errors in Table 1 mistakenly carried forward solely by the production team that handled this article.

The correct presentation of Table 1 can be viewed ahead in this correction article.

**Table 1:** Comparison of WHO-HAEM4R, ICC and WHO-HAEM5 classification mature B-cell lymphomas, leukemias and lymphoproliferative disorders

WHO-HAEM4R	International Consensus Conference (ICC)	WHO-HAEM5
Mature B-cell lymphomas, leukemias and lymphoproliferative disorders		
**Chronic lymphocytic leukemia, small lymphocytic lymphoma, B-cell prolymphocytic leukemia and monoclonal B lymphocytosis**		
Chronic lymphocytic leukemia/small lymphocytic lymphoma (CLL/SLL)	CLL/SLL	CLL/SLL
Monoclonal B-cell lymphocytosis (MBL) - CLL type - Non-CLL type	MBL - CLL type - Non-CLL type	MBL - CLL type - Non-CLL type
B-Prolymphocytic leukemia (B-PLL)	B-PLL	Category removed
**Splenic B cell lymphomas/leukemias**		
Hairy cell leukemia	Hairy cell leukemia	Hairy cell leukemia
Splenic MZL (SMZL)	SMZL	SMZL
*Splenic B-cell leukemia lymphoma*,* unclassifiable*	*Splenic B-cell leukemia lymphoma*,* unclassifiable*	
- *Splenic diffuse red pulp small B-cell lymphoma* - *Hairy cell leukemia-variant*	- *Splenic diffuse red pulp small B-cell lymphoma* - *Hairy cell leukemia-variant*	Splenic diffuse red pulp small B-cell lymphoma Splenic B-cell lymphoma/leukemia with prominent nucleoli
**Lymphoplasmacytic lymphoma**		
Lymphoplasmacytic lymphoma (LPL)	LPL	LPL
**Marginal zone lymphomas**		
Extranodal marginal zone lymphoma of mucosa-associated lymphoid tissue (EMZL)	EMZL	EMZL
Primary cutaneous MZL (included under EMZL)	Primary cutaneous marginal zone lymphoproliferative disorder	Primary cutaneous MZL
Nodal MZL *-*Pediatric MZL	Nodal MZL -Pediatric nodal MZL	Nodal MZL
		Pediatric nodal MZL
**Follicular lymphoma**		
Follicular lymphoma (FL) (FL, grades 1–3 A, 3B) - In situ follicular neoplasia - Duodenal-type FL - Testicular FL Pediatric type FL Primary cutaneous follicle center lymphoma Also recognized are rare, largely diffuse cases, often inguinal, lacking BCL2 rearrangement and expressing CD23 with loss at 1p36.	FL (FL, grade 1–3 A, 3B) - In situ follicular neoplasia - Duodenal-type FL *BCL2-R* negative, CD23 + follicle center lymphoma Primary cutaneous follicle center lymphoma Pediatric type FL Testicular FL	FL - Classic FL (cFL; grading [1–3 A] is optional) - Follicular large B-cell lymphoma - FL with unusual cytologic features (blastoid or large centrocyte variant cytologic features) - FL with predominantly diffuse growth pattern In situ follicular B-cell neoplasm Duodenal-type FL Pediatric type FL Primary cutaneous follicle center lymphoma
**Mantle cell lymphoma**		
Mantle cell lymphoma (MCL) - In situ MCL - Non-nodal MCL	MCL - In situ MCL - Leukemic non-nodal MCL	MCL - In situ mantle cell neoplasm - Leukemic non-nodal MCL
**Large B-cell lymphomas**		
Diffuse large B-cell lymphoma, not otherwise specified (DLBCL, NOS) *Molecular subtypes* - Germinal center B-cell subtype - Activated B-cell subtype *Morphological variants* - Centroblastic - Immunoblastic - Anaplastic - Other rare variants	DLBCL, NOS - Germinal center B-cell type - Activated B-cell type	DLBCL, NOS - Germinal center B-cell subtype - Activated B-cell subtype - Centroblastic subtype - Immunoblastic subtype - Anaplastic subtype - DLBCL with *MYC* and *BCL6* rearrangements
T-cell/histocyte rich large B-cell lymphoma (TCHRLBCL)	TCHRLBCL	TCHRLBCL
High grade B-cell lymphoma (HGBCL) with *MYC* and *BCL2* and/or *BCL6* rearrangement	HGBL with *MYC* and *BCL2* rearrangement	DLBCL/HGBL with *MYC* and *BCL2* rearrangement
	*HGBL with MYC and BCL6 rearrangement*	(see DLBCL, NOS)
ALK + LBCL	ALK + LBCL	ALK + LBCL
Large B-cell lymphoma with IRF4 rearrangement	Large B-cell lymphoma with *IRF4* rearrangement	Large B-cell lymphoma with *IRF4* rearrangement
*Burkitt-like lymphoma with 11q aberration*	*Large B-cell lymphoma with 11q aberration*	High-grade B-cell lymphoma with 11q aberration
Lymphomatoid granulomatosis	Lymphomatoid granulomatosis	Lymphomatoid granulomatosis
EBV + DLBCL, NOS	EBV + DLBCL, NOS	EBV + DLBCL
DLBCL associated with chronic inflammation (DLBCL-CI) - Fibrin-associated diffuse large B-cell lymphoma	DLBCL-CI - Fibrin-associated diffuse large B-cell lymphoma	DLBCL-CI Fibrin-associated large B-cell lymphoma
HHV8-negative effusion-based lymphoma (noted in differential diagnosis of PEL; not a distinct entity)	*HHV8 and EBV-negative primary effusion-based lymphoma*	Fluid overload-associated large B-cell lymphoma
Plasmablastic lymphoma (PBL)	PBL	PBL
Primary DLBCL of the CNS	Primary DLBCL of the CNS	Primary LBCL of immune-privileged sites (includes CNS, vitreoretinal, testis)
	Primary DLBCL of the testis	
Primary cutaneous diffuse large B-cell lymphoma, leg type	Primary cutaneous diffuse large B-cell lymphoma, leg type	Primary cutaneous diffuse large B-cell lymphoma, leg type
Intravascular large B-cell lymphoma	Intravascular large B-cell lymphoma	Intravascular large B-cell lymphoma
Primary mediastinal large B-cell lymphoma (PMBCL)	PMBCL	PMBCL
B-cell lymphoma, unclassifiable with features intermediate between DLBCL and classic HL	Mediastinal gray zone lymphoma	Mediastinal grey zone lymphoma
HGBCL, NOS	HGBCL, NOS	HGBCL, NOS -HGBCL with *MYC* and *BCL6* rearrangements
**Burkitt lymphoma** (BL)	BL	BL
**KSHV/HHV8-associated lymphoproliferative disorders and lymphomas**		
Primary effusion lymphoma (PEL) Subtype: extracavitary PEL	PEL Subtype: extra-cavitary PEL	PEL Subtype: extracavitary PEL
*HHV-8 positive DLBCL*,* NOS*	HHV-8 positive DLBCL, NOS	KSHV/HHV8 positive DLBCL
HHV-8 positive germinotropic lymphoproliferative disorder	HHV-8 positive germinotropic lymphoproliferative disorder	KSHV/HHV8 positive germinotropic lymphoproliferative disorder
	Multicentric Castleman Disease	KSHV/HHV8-associated multicentric Castleman Disease (included under Tumour-like lesions with B-cell predominance)
**Lymphoid proliferations and lymphomas associated with immune deficiency and dysregulation**		
Lymphoproliferative diseases associated with primary immune disorders Lymphomas associated with HIV infection Post-transplant lymphoproliferative disorders (PTLDs) - Nondestructive (including plasmacytic hyperplasia PTLD, infectious mononucleosis (IM) PTLD, florid follicular hyperplasia (FFH) PTLD) - Polymorphic PTLD - Monomorphic PTLD (classified based on the lymphoma type to which they best correspond) - Classic Hodgkin lymphoma PTLD Other iatrogenic immunodeficiency-associated LPDs	PTLDs - Plasmacytic hyperplasia PTLD - IM PTLD - FFH PTLD - Polymorphic PTLD - Monomorphic PTLD (B-, T/NK-cell types) - Classic Hodgkin lymphoma PTLD Other iatrogenic immunodeficiency-associated LPDs	Hyperplasias arising in immune deficiency/dysregulation Polymorphic LPDs arising in immune deficiency/ dysregulation EBV + mucocutaneous ulcer Lymphomas arising in immune deficiency/dysregulation Inborn error of immunity-associated lymphoid proliferations and lymphoma *New nomenclature: 1) histologic diagnosis according to accepted criteria* *2) Presence or absence of oncogenic virus(es)* *3) Clinical setting/ immunodeficiency background*
*EBV + mucocutaneous ulcer*	EBV + mucocutaneous ulcer	EBV + MCU (included in the category above)
	EBV + polymorphic B-cell LPD, NOS	Polymorphic LPDs arising in immune deficiency/ dysregulation (included above)

Italics – provisional entity

